# Cocaine-Induced Ocular Toxicity

**DOI:** 10.3390/diseases14080274

**Published:** 2026-07-30

**Authors:** Alessandra Pizzo, Marco Zeppieri, Filippo Marano, Alessandro Vasco, Corrado Pizzo, Antonio M. Vicari, Fabiana D’Esposito, Caterina Gagliano, Francesco Cappellani

**Affiliations:** 1 Unità Operativa Complessa di Oftalmologia, Azienda Ospedaliera per l’Emergenza “Cannizzaro”, 95026 Catania, Italy; 2 Department of Ophthalmology, University Hospital of Udine, 33100 Udine, Italy; 3 Department of Medicine, Surgery and Health Sciences, University of Trieste, 34129 Trieste, Italy; 4 Unità Operativa Complessa di Oftalmologia, Azienda Ospedaliera Sant’Elia, 93100 Caltanissetta, Italy; 5 Istituto di Oftalmologia, Università degli Studi di Catania, 95123 Catania, Italy; 6 Imperial College Ophthalmic Research Group (ICORG) Unit, Imperial College, 153-173 Marylebone Rd., London NW1 5QH, UK; 7 Department of Medicine and Surgery, University of Enna “Kore”, Piazza dell’Università, 94100 Enna, Italyfrancesco.cappellani@unikore.it (F.C.); 8 Mediterranean Foundation “G.B. Morgagni”, 95125 Catania, Italy

**Keywords:** cocaine-related disorders, oxidative stress, corneal epithelial defects, retinal vascular occlusion, ischemic optic neuropathy, substance-related disorders

## Abstract

Cocaine-induced ocular toxicity is an increasingly acknowledged but often underestimated clinical condition that includes a wide range of eye-related symptoms. The extensive recreational use of cocaine, together with its powerful sympathomimetic and vasoconstrictive effects, leads to various ocular problems that can impact all anatomical components of the eye. This narrative review aims to deliver a thorough and current synthesis of the existing research on the epidemiology, pathophysiology, clinical symptoms, and therapy of cocaine-related ocular illness. Evidence suggests that cocaine-induced ocular damage involves vascular, local toxic, neuronal, and immunological processes. Reported manifestations in the literature include the ocular surface, optic nerve, posterior segment, orbit, and adnexal tissues. Novel imaging techniques have revealed subclinical retinal microvascular changes in chronic users, suggesting a continuum from initial vascular dysregulation to manifest ischemic injury. Notwithstanding increased awareness, the existing evidence is constrained by heterogeneity, small sample sizes, and a prevalence of case reports. A comprehensive understanding of the pathophysiological mechanisms and long-term ocular effects is crucial for enhancing diagnostic precision, directing management, and informing preventive measures. Enhanced multidisciplinary collaboration between ophthalmologists and addiction experts is essential to tackle this intricate and dynamic clinical dilemma.

## 1. Introduction

Cocaine remains one of the most clinically consequential illicit stimulants worldwide and continues to be associated with substantial systemic morbidity, particularly involving the cardiovascular, neurologic, and vascular systems. Although these systemic effects are well recognized, ocular complications have received comparatively less focused attention, despite the fact that the eye may be affected through both local toxic effects and broader vascular or inflammatory pathways. Historically, cocaine also occupies a unique place in ophthalmology, as it was among the earliest agents introduced as a topical ocular anesthetic because of its sodium-channel blocking properties. At the same time, its systemic pharmacologic activity is primarily mediated through inhibition of dopamine, norepinephrine, and serotonin reuptake, producing a marked sympathomimetic state that underlies many of its toxic effects [1,2].

From an ophthalmic perspective, cocaine-related disease is notable for its breadth rather than for a single characteristic presentation. Clinical manifestations range from retinal vascular occlusions, chorioretinal ischemia, optic neuropathy, orbital inflammation, and sinonasal destructive lesions with orbital extension to punctate keratopathy, persistent epithelial defects, microbial keratitis, and neurotrophic corneal disease. In selected cases, ocular pathology may also reflect the contribution of adulterants present in illicit cocaine preparations, particularly levamisole, which has been associated with immune-mediated inflammatory syndromes and vasculitic phenomena [3,4].

The mechanisms responsible for these manifestations appear to be heterogeneous and frequently overlapping. Vascular dysregulation is likely a major component, with cocaine exposure having been associated with vasoconstriction, endothelial dysfunction, oxidative stress, platelet activation, and thrombosis. Repeated exposure has also been associated with ocular surface damage related to corneal hypoesthesia, altered tear function, and epithelial compromise, especially in crack cocaine users or in patients with direct ocular exposure [5,6]. More recently, advances in ocular imaging have added a further layer of complexity to this field. Isolated reports and small observational studies have suggested that chronic cocaine use may be associated with subclinical retinal vascular and structural abnormalities, including changes in retinal vascular caliber, enlargement of the foveal avascular zone, and thinning of the retinal nerve fiber layer [7,8,9]. Recognition of cocaine-related ocular toxicity remains challenging in routine practice. Presentations are often nonspecific, patient disclosure of substance use may be incomplete, and the resulting ocular findings can mimic more common ischemic, infectious, inflammatory, autoimmune, or compressive disorders.

Despite the growing number of publications on this topic, the evidence base remains fragmented and is derived predominantly from case reports, small case series, and limited cross-sectional or retrospective studies. The present narrative review summarizes current evidence on cocaine-induced ocular toxicity, with particular attention to pathophysiological mechanisms, clinical manifestations across different ocular structures, diagnostic considerations, and current management challenges, while also highlighting the principal limitations of the existing evidence and priorities for future research.

## 2. Materials and Methods

This narrative review aims to provide a thorough and up-to-date synthesis of the literature on cocaine-induced ocular damage, incorporating clinical, translational, and mechanistic findings. A targeted literature search was conducted to identify pertinent papers published from January 1990 to February 2026, with seminal works before this timeframe included as appropriate to contextualize fundamental concepts. The search approach integrated Medical Subject Headings with free-text terms such as “cocaine,” “ocular toxicity,” “eye disease,” “retinal occlusion,” “retina,” “cornea,” “optic neuropathy,” “glaucoma,” and “vasculopathy.” Boolean operators were utilized to enhance the search and guarantee thorough coverage of the subject. The reference lists of selected papers were manually examined to identify additional pertinent publications not included in the initial database search.

The inclusion criteria were original clinical investigations, case reports, observational cohorts, case–control studies, cross-sectional analyses, and translational or experimental research that offered significant insights into the causes, clinical manifestations, or management of cocaine-related ocular disease. Only articles published in English and indexed in PubMed were deemed eligible for inclusion.

Data extraction was conducted qualitatively, emphasizing critical aspects such as pathophysiological causes, anatomical patterns of ocular involvement, clinical presentation, diagnostic results, and therapeutic strategies. Due to the variability in study designs and outcomes, a formal quantitative meta-analysis proved impractical. Findings were summarized narratively, highlighting consistency among research, biological plausibility, and clinical significance.

## 3. Pathophysiological Mechanisms in Cocaine-Induced Ocular Toxicity

Cocaine-related ocular toxicity appears to be multifactorial, involving overlapping vascular, thrombotic, neural, ocular surface, and, in selected cases, immune-mediated mechanisms. Because cocaine inhibits the reuptake of norepinephrine, dopamine, and serotonin, its systemic effects are characterized by marked sympathomimetic stimulation, which promotes vasoconstriction and contributes to many of its toxic manifestations [1,5,10].

### 3.1. Vascular Dysregulation and Ischemic Injury

Vascular dysregulation is likely the best-supported mechanism underlying cocaine-related ocular damage, particularly in posterior-segment disease. Cocaine has been associated with vasoconstriction, endothelial dysfunction, oxidative stress, inflammatory vascular signaling, and impaired vascular homeostasis, changes that may compromise ocular perfusion and predispose to ischemic injury [1,5,11,12]. The retina is especially susceptible to such abnormalities because of its high metabolic demand and limited collateral circulation [13,14].

In addition to vasoconstriction, cocaine may also promote thrombotic complications through platelet activation, aggregation, and thrombus formation, thereby further increasing the risk of vascular occlusion [11,13,15]. Case reports have described cocaine-associated chorioretinal infarction and outer retinal ischemic injury, including acute macular neuroretinopathy, suggesting that vascular compromise may extend beyond the retinal circulation alone [16,17]. Acute systemic hypertension and hemodynamic fluctuations induced by cocaine may further aggravate these ischemic events, particularly in susceptible individuals [5,10]. Although recurrent vasospasm and microvascular occlusion have been proposed as contributors to repeated injury, longitudinal evidence for a progressive chronic retinal ischemic process remains limited [13]. Controlled in vivo studies provide additional mechanistic support for cocaine-induced vascular dysregulation. In rats, multimodal cortical imaging demonstrated an immediate reduction in cerebral blood flow and hemoglobin oxygenation following cocaine administration, followed by a delayed vascular overshoot, while neuronal calcium activity remained elevated. Complementary studies in mice found that cocaine-induced vasoconstriction was associated with a more prolonged increase in astrocytic calcium signaling. Pharmacologic inhibition of astrocytic activity attenuated the vascular constriction, suggesting that astrocytes may contribute to the persistence of cocaine-induced cerebrovascular dysfunction [18,19].

These controlled investigations have shown cocaine-induced vascular and neurovascular anomalies in the cerebral circulation, not the ocular. Extrapolation is limited by cerebral, retinal, choroidal, and optic-nerve vascular bed differences. The data support the biological possibility that cocaine-induced vasoconstriction, neurovascular coupling, and tissue oxygenation may cause ocular ischemia episodes. To directly confirm these mechanisms, retinal and choroidal perfusion experiments are needed.

### 3.2. Neural and Retinal Dysfunction

Disturbances in color vision, particularly blue-yellow discrimination, have been reported in cocaine and stimulant users, and retinal dopaminergic dysfunction has been proposed as one possible contributing mechanism [20,21]. However, direct ocular neurotoxicity remains less firmly established than vascular injury, and current evidence is based largely on observational studies and case reports rather than on direct mechanistic confirmation in human ocular tissues [3,22].

Structural studies have also suggested possible neural involvement. Optical coherence tomography has shown thinner peripapillary retinal nerve fiber layer measurements in cocaine users, raising the possibility of inner retinal or optic nerve pathway abnormalities [9]. In addition, reports have described cocaine-related optic nerve injury, including posterior ischemic optic neuropathy and toxic ischemic optic neuropathy, indicating that neural dysfunction may coexist with vascular damage in some patients [12,23,24]. Controlled animal studies also provide evidence of acute cocaine-related disruption of visual sensory-motor function. In mice, intraperitoneal cocaine increased spontaneous eye-movement frequency at lower doses, whereas higher doses suppressed visually evoked optokinetic responses while inducing stereotyped spontaneous eye movements. These findings demonstrate a dose-dependent effect of cocaine on oculomotor circuitry, although they do not establish direct retinal or optic nerve toxicity [25].

### 3.3. Ocular Surface Injury

The ocular surface represents another important target of cocaine-related injury. Repeated exposure may reduce corneal sensitivity and impair tear function, likely as a consequence of cocaine’s local anesthetic properties and its effects on corneal trophic support [2,6]. This may increase epithelial exposure and susceptibility to mechanical injury, particularly in the setting of eye rubbing, reduced blink function, and environmental exposure associated with crack cocaine use or direct ocular contact [6,26].

Clinical studies support an association between cocaine use and ocular surface damage [6], ranging from punctate keratopathy to ulceration and, in severe cases, perforation, as described in reports of crack cocaine-associated corneal complications [26,27]. Barrier dysfunction may also predispose to secondary infection. Thus, ocular surface toxicity likely reflects both direct local injury and secondary complications arising from impaired corneal sensation and epithelial instability [3,6].

### 3.4. Immune-Mediated and Adulterant-Related Mechanisms

A smaller but clinically important subset of cocaine-related ocular disease may be mediated by immune dysregulation, particularly in the setting of adulterated cocaine. Levamisole, a common adulterant in illicit cocaine, has been associated with ANCA-positive vasculitic syndromes and other immune-mediated inflammatory reactions [4,28]. Ocular manifestations appear to be uncommon and are documented mainly in case reports, but reported findings include cicatrizing conjunctivitis, ocular cicatricial pemphigoid-like disease, orbital inflammation, and severe sinonasal-orbital destructive pathology with possible optic nerve involvement [4,28,29]. Illicit cocaine may contain several pharmacologically active adulterants whose toxic effects can complicate attribution of ocular and systemic findings to cocaine alone. The composition of street cocaine varies geographically and over time, and most adulterants have not been specifically investigated in ocular models. Table 1 summarizes the principal clinically relevant adulterants identified in cocaine samples, distinguishing their established independent toxicity from potential additive effects when combined with cocaine [4,28,30,31,32].

These observations suggest that, in selected patients, ocular disease may reflect not only the direct effects of cocaine itself but also an inflammatory response triggered by contaminants. Differentiating direct cocaine toxicity from adulterant-related disease may be difficult in practice because ischemic, inflammatory, and destructive features can coexist. The presence and prevalence of adulterants vary by region, period, and drug formulation. Except for levamisole, evidence directly connecting individual cocaine adulterants to defined ocular disease is limited; for most agents, ocular relevance is indirect or biologically plausible rather than clinically established. Reported toxicity may reflect the adulterant alone, cocaine alone, additive effects, or interactions with other substances. This overlap broadens the pathophysiological spectrum of cocaine-related ocular disease and may have implications for both diagnostic work-up and treatment.

## 4. Clinical Manifestations of Cocaine Ocular Toxicity

The clinical manifestations of cocaine-related ocular toxicity are diverse and range from relatively limited ocular surface disease to severe, vision-threatening involvement of the retina, optic nerve, and orbit. The observed phenotype may vary according to route of administration, chronicity of use, and the presence of adulterants. Some patients present with atypical or rapidly progressive ocular findings, including presentations in younger individuals or in those without conventional vascular risk factors. The temporal correlation between cocaine consumption and the start of symptoms may yield significant diagnostic insights, but it is not consistently well-defined. Recent literature, including a thorough examination of ocular symptoms linked to cocaine use, emphasizes the extensive clinical involvement, necessitating a systematic and anatomically focused evaluation strategy [3]. For practical purposes, the reported manifestations can be grouped into anterior segment, posterior segment, neuro-ophthalmic, and orbital/adnexal patterns, although overlap between these categories may occur.

Involvement of the anterior segment is one of the most commonly documented signs, mostly due to direct toxicity and neurotrophic processes affecting the ocular surface. Patients frequently exhibit symptoms including ocular discomfort, photophobia, foreign body sensation, and diminished visual acuity, which may initially seem vague. Nevertheless, meticulous analysis frequently reveals distinctive findings, such as punctate epithelial erosions, persistent epithelial abnormalities, and, in more advanced cases, corneal ulceration. Crack eye syndrome exemplifies anterior region toxicity, with a spectrum of corneal damage linked to cocaine inhalation or direct ocular contact [26]. Reduced corneal sensitivity, reduced tear production and tear-film instability may further aggravate ocular-surface irritation and compromise epithelial integrity. Secondary microbial keratitis may complicate the clinical course and, in severe cases, require intensive antimicrobial therapy. Advanced disease may progress to corneal perforation and, occasionally, surgical intervention [6,26,27].

Involvement of the posterior segment is primarily marked by vascular pathology and is one of the most significant threats to vision associated with cocaine-induced ocular toxicity. Patients may exhibit abrupt, painless vision loss, scotomas, or visual field impairments, frequently without accompanying systemic comorbidities. Retinal vascular occlusions, encompassing central and branch retinal artery occlusions as well as central retinal vein occlusion, are frequently documented complications [6,33]. Fundoscopic examination may disclose retinal whitening, hemorrhages, venous dilatation, and cotton wool patches, contingent upon the particular vascular incident. Moreover, instances of chorioretinal infarction and acute macular neuroretinopathy have been documented, further exemplifying the range of ischemic damage impacting the posterior segment [16,17]. Optical coherence tomography and angiography furnish critical diagnostic insights, uncovering structural and microvascular changes that may remain undetected during clinical evaluation alone [7,8].

Neuro-ophthalmic manifestations, although less commonly reported than corneal or retinal vascular complications, represent an important part of the cocaine-related ocular spectrum because they may result in substantial and sometimes permanent visual morbidity. Reported findings include optic neuropathy, anisocoria, ophthalmoplegia, and visual dysfunction related to involvement of either the peripheral or central nervous system. Cocaine-related optic neuropathy has been described with features such as reduced visual acuity, relative afferent pupillary defect, and, depending on the site and mechanism of injury, optic disk swelling or subsequent pallor [12,33,34,35,36].

Cocaine use has been associated with cerebrovascular events, including brainstem ischemia, which may lead to neuro-ophthalmic deficits such as internuclear ophthalmoplegia. Anisocoria may also occur because of cocaine’s sympathomimetic effect on the iris dilator muscle, particularly after inadvertent ocular exposure. In addition, rare reports suggest that cocaine abuse may unmask or exacerbate disorders such as myasthenia gravis, thereby broadening the neuro-ophthalmic spectrum [33,34,35,36].

Orbital and adnexal involvement represents an important, though less common, component of cocaine-related ocular disease and is most often associated with prolonged intranasal use and local sinonasal tissue destruction. Patients may present with periorbital pain, edema, diplopia, and reduced vision, reflecting extension of inflammatory or destructive disease from the nasal cavity and paranasal sinuses into the orbit. Cocaine-induced midline destructive lesions are a recognized complication characterized by progressive necrosis of nasal and sinus tissues, which in severe cases may extend into the orbit and produce substantial structural damage [37,38,39].

These lesions may be associated with secondary infection, inflammation, and mass effect, leading to complications such as orbital cellulitis, dacryocystitis, and orbital apex syndrome. In selected cases, immune-mediated mechanisms related to adulterants such as levamisole may also contribute to inflammatory or vasculitic ocular manifestations, further complicating the clinical picture [40,41].

Glaucoma-related manifestations have been reported in association with cocaine use, although the evidence is much stronger for acute angle-closure events than for chronic glaucoma risk. Acute angle-closure glaucoma has been described after intranasal or topical cocaine exposure, most likely because cocaine-induced mydriasis can precipitate pupillary-block angle closure in anatomically predisposed individuals [42,43,44]. Conversely, observational data have suggested an association between cocaine use and open-angle glaucoma, although this relationship remains incompletely characterized and the underlying mechanisms are uncertain [45]. Table 2 summarizes the primary ocular outcomes and their proposed underlying mechanisms, highlighting the multifaceted nature of cocaine-induced ocular toxicity.

## 5. Clinical Assessment and Diagnostic Procedures

The diagnosis of cocaine-related ocular toxicity requires a systematic clinical approach that integrates careful history-taking, comprehensive ophthalmic examination, and the judicious use of imaging when indicated. Identification of cocaine exposure is a key part of the diagnostic process, but substance use may be underreported because of stigma, reluctance to disclose illicit drug use, or failure to recognize its relevance to ocular disease. Clinicians must uphold a heightened level of skepticism, especially when faced with atypical manifestations such as retinal vascular occlusions, optic neuropathy, or severe ocular surface disease in younger patients lacking traditional risk factors. Precise and nonjudgmental inquiries about substance use are crucial, as the timing of cocaine intake in relation to symptom start may yield significant diagnostic insights.

A comprehensive eye examination is fundamental to clinical assessment and must encompass evaluation of visual acuity, intraocular pressure, pupillary reactions, extraocular motility, and slit-lamp biomicroscopy. Special attention should be paid to indicators of ocular surface impairment, including epithelial abnormalities, diminished corneal sensitivity, and tear film instability, which may suggest neurotrophic keratopathy. Fundoscopic examination is crucial for detecting posterior segment pathology, such as retinal hemorrhages, vascular occlusions, and optic disk anomalies. In cases of potential neuro-ophthalmic involvement, visual field testing and color vision evaluation may provide additional functional insights. Anisocoria or ophthalmoplegia necessitates additional assessment to rule out central nervous system involvement, including imaging when appropriate.

Advanced imaging techniques play an important role in the evaluation of cocaine-related ocular disease. Optical coherence tomography can identify structural abnormalities involving the retina and optic nerve, including retinal nerve fiber layer thinning and, in selected cases, disruption of retinal architecture. Optical coherence tomography angiography has also been used to demonstrate retinal microvascular abnormalities, including enlargement of the foveal avascular zone, while observational studies have reported early retinal vascular changes in cocaine users [7,8,9]. Fluorescein angiography plays an important role in the evaluation of retinal vascular occlusions and ischemic retinal changes by providing information on retinal perfusion and leakage. In patients with suspected orbital or optic nerve involvement, computed tomography and magnetic resonance imaging are particularly useful for defining inflammation, mass effect, and tissue destruction associated with cocaine-induced midline lesions or chronic sinonasal-orbital disease. The integration of structural and vascular imaging can improve clinical characterization of cocaine-related ocular disease [37,38,46].

Toxicology screening can provide objective evidence of recent cocaine exposure, although its diagnostic utility is limited by specimen-dependent detection windows and by variability related to dose, route of administration, and chronicity of use. Evaluation of systemic comorbidities, including cardiovascular risk factors and concurrent substance use, is also important for a comprehensive assessment. Laboratory findings should be interpreted within the broader clinical context, as many abnormalities may be nonspecific or difficult to interpret in isolation [3].

Differential diagnosis is a crucial component of the assessment process, as most cocaine-induced ocular diseases resemble other ophthalmic and systemic disorders. Retinal vascular occlusions in young patients may necessitate evaluation for hypercoagulable conditions, autoimmune diseases, or viral causes, whereas optic neuropathy may indicate the possibility of demyelinating diseases or compressive lesions. Prompt identification is essential, as immediate care can avert irreversible vision impairment and enhance patient outcomes. Nonetheless, the absence of defined diagnostic criteria and the variability of clinical presentations continue to pose considerable obstacles. Subsequent research should focus on developing more precise diagnostic frameworks and identifying biomarkers to enhance early diagnosis and risk classification.

## 6. Discussion

Cocaine-induced ocular toxicity is a complicated clinical condition resulting from the interplay of vascular, neurotoxic, inflammatory, and surface-related mechanisms, leading to a diverse range of symptoms. This analysis highlights the significance of identifying cocaine use as a notable and sometimes overlooked risk factor in patients exhibiting unusual ocular disease, especially in younger persons lacking conventional systemic comorbidities. Clinically, a notable feature is the extensive anatomical distribution of ocular involvement. This diversity in presentation frequently leads to diagnostic delays or misattribution to more prevalent causes, thereby increasing the risk of irreversible vision impairment. The presence of multiple disease processes in a single patient complicates clinical evaluation and requires a thorough, integrative diagnostic strategy. The clinical significance of these results necessitates increased vigilance among ophthalmologists and other healthcare professionals, as early detection of cocaine-related ocular conditions may substantially affect therapeutic approaches and patient guidance.

The relationship between subclinical retinal abnormalities and clinically overt ischemic events remains particularly uncertain. Enlargement of the foveal avascular zone, alterations in retinal vascular caliber, and thinning of the retinal nerve fiber layer have been reported in cocaine users without acute vascular occlusion. In contrast, retinal artery occlusion, chorioretinal infarction, acute macular neuroretinopathy, and ischemic optic neuropathy have generally been described as abrupt clinical events. Although the imaging findings may reflect early or cumulative vascular and neural injury, current studies are predominantly cross-sectional and involve populations that differ from those represented in ischemic case reports. Consequently, it remains unknown whether these subclinical abnormalities predict subsequent vascular events, represent markers of exposure, or reflect independent effects influenced by comorbidities and concurrent substance use. Controlled experimental models strengthen the biological plausibility of cocaine-induced neurovascular dysfunction while also helping to separate direct drug effects from the confounding factors present in clinical reports. In rats, cocaine produced an immediate reduction in cerebral blood flow and hemoglobin oxygenation, followed by a delayed vascular overshoot despite sustained neuronal activation. Complementary mouse studies demonstrated that prolonged cocaine-induced vasoconstriction was associated with persistent astrocytic calcium signaling and was attenuated by pharmacologic inhibition of astrocytic activity. These findings support the possibility that cocaine can produce both rapid and sustained disturbances in neurovascular regulation. However, because these experiments examined cerebral rather than retinal or choroidal circulation, their relevance to ocular ischemia should be regarded as mechanistic support rather than direct evidence of ocular vascular injury. Moreover, controlled experimental models may help clarify some of the variability observed in human cocaine toxicity. In mice with combined butyrylcholinesterase and plasma carboxylesterase deficiency, cocaine-induced toxic effects, including nonspecific ocular manifestations, were markedly prolonged compared with control animals, suggesting that differences in cocaine metabolism may influence the severity and duration of toxicity, although specific ocular tissue injury was not characterized [47]. A further diagnostic challenge is distinguishing the direct effects of cocaine from immune-mediated disease associated with adulterants such as levamisole. A predominantly ischemic phenotype temporally related to cocaine exposure, including retinal vascular occlusion, chorioretinal ischemia, acute angle closure, or local ocular-surface injury, may be more consistent with direct pharmacologic or vascular toxicity. Conversely, destructive sinonasal disease, cicatrizing conjunctivitis, orbital inflammation, cutaneous vasculitic lesions, neutropenia, or atypical ANCA positivity should raise suspicion of an adulterant-associated immune process. These categories are not mutually exclusive, however, because vascular, inflammatory, infectious, and destructive mechanisms may coexist in the same patient. Clinical interpretation should therefore integrate the route and timing of exposure, systemic manifestations, imaging findings, and targeted laboratory investigations rather than rely on a single diagnostic marker.

Notwithstanding the expanding body of literature, certain significant limitations must be recognized when analyzing the existing evidence. A considerable amount of published data originates from case reports and small case series, which automatically limits generalizability and increases the potential for reporting bias. Extensive observational studies remain very limited, and the existing ones often rely on retrospective methods and administrative databases, which may lack comprehensive clinical information and overlook confounding variables. The variability of study subjects, variations in cocaine composition, and disparities in administration routes further complicate the interpretation of findings. The function of adulterants, although increasingly acknowledged, is inadequately quantified in several studies, complicating the isolation of the precise contributions of cocaine and its impurities. The methodological challenges highlight the need for more rigorous, standardized research methodologies in this domain.

Additional studies are essential to clarify the natural history of cocaine-induced ocular damage and to ascertain whether subclinical alterations seen with advanced imaging proceed to clinically relevant disease. The advancement of biomarkers, encompassing imaging-based and molecular indications, may enable early diagnosis and risk stratification. Experimental research examining the cellular and molecular impacts of cocaine on ocular tissues may yield significant insights into underlying mechanisms. Interdisciplinary collaboration among ophthalmologists, neurologists, toxicologists, and addiction experts will be essential to advancing research and therapeutic care. Despite considerable advances in delineating the clinical spectrum and underlying mechanisms, significant gaps persist in our understanding of disease development, appropriate therapeutic approaches, and long-term outcomes. Enhanced knowledge and a comprehensive understanding of cocaine-related ocular disease can improve patient care, diminish visual morbidity, and support broader public health initiatives to alleviate the effects of substance addiction.

## 7. Conclusions

Cocaine-related ocular toxicity comprises a broad and clinically important spectrum of disease involving multiple ocular structures. The available evidence suggests that these manifestations arise through multiple, often overlapping mechanisms. Because presentations may be atypical and may occur in younger individuals without conventional risk factors, cocaine exposure should be considered in the differential diagnosis of unusual ocular disease patterns.

Despite growing recognition of this entity, the current literature remains limited by methodological heterogeneity, small sample sizes, and a predominance of case reports and retrospective studies. As a result, many aspects of disease mechanisms, prognosis, and optimal management remain incompletely defined. Greater awareness, careful clinical assessment, and interdisciplinary collaboration are essential to improve recognition and patient care. Future studies should focus on better phenotypic characterization, longitudinal outcomes, and the development of more robust diagnostic and therapeutic frameworks for cocaine-related ocular disease.

## Figures and Tables

**Table 1:** Principal pharmacologically active adulterants reported in illicit cocaine and their potential clinical relevance [4,28,30,31,32].

Adulterant/Contaminant	Main Independent Toxicity	Potential Interaction with Cocaine	Ocular Relevance
Levamisole	Agranulocytosis, retiform purpura, ANCA-associated vasculitis, leukoencephalopathy	May add immune-mediated and vascular injury to cocaine-related toxicity	Best-supported ocular adulterant; reported associations include cicatrizing conjunctivitis, pemphigoid-like disease, orbital inflammation, and sinonasal-orbital destruction
Local anesthetics (lidocaine, benzocaine, procaine)	Neurologic and cardiac toxicity; methemoglobinemia with some agents	May increase sodium-channel–blocking and local anesthetic effects	Direct ocular exposure may aggravate corneal hypoesthesia or epithelial injury, but evidence is limited
Caffeine	Tachycardia, hypertension, agitation, arrhythmia	May enhance sympathomimetic and cardiovascular stress	No direct ocular toxicity established; possible indirect vascular relevance
Phenacetin	Renal, hematologic, and carcinogenic toxicity	May add systemic toxicity and impair oxygen delivery	No specific ocular effect established
Synthetic opioids	Respiratory depression, hypoxia, coma	May produce mixed stimulant–opioid toxicity and worsen hypoxic injury	Ocular effects are mainly secondary to systemic hypoxia rather than direct toxicity

**Table 2:** Ocular complications linked to cocaine use.

Ocular Structure/Pattern	Main Clinical Manifestations	Proposed Mechanisms
Anterior segment/ocular surface	Neurotrophic keratopathy, persistent epithelial defects, microbial keratitis, corneal ulceration/perforation	Local anesthetic effect, corneal hypoesthesia, tear-film impairment, epithelial instability, secondary infection, cocaine-induced mydriasis with pupillary block in predisposed eyes
Posterior segment ischemic	Retinal vascular occlusions, chorioretinal infarction, acute macular neuroretinopathy	Vasoconstriction, endothelial dysfunction, thrombosis, retinal and choroidal ischemia
Neuro-ophthalmic	Optic neuropathy, dyschromatopsia, visual field defects, anisocoria, ophthalmoplegia	Ischemic injury, inflammatory involvement, pharmacologic mydriasis, mixed toxic ischemic or central neurologic mechanisms
Orbital/sinonasal	Orbital inflammation, orbital cellulitis, dacryocystitis, orbital apex syndrome	Sinonasal tissue destruction, secondary infection/inflammation
Immune-mediated	Cicatrizing conjunctivitis, OCP-like disease, vasculitic ocular inflammation	Levamisole-associated ANCA-mediated immune dysregulation
Glaucoma-related	Acute angle-closure glaucoma; possible association with open-angle glaucoma	Mydriasis-induced angle closure; uncertain chronic mechanisms

Abbreviations: OCP, ocular cicatricial pemphigoid; ANCA, antineutrophil cytoplasmic antibody.

## Data Availability

No new data were created or analyzed in this study. Data sharing is not applicable to this article.
